# Efficient Authentication Scheme for 5G-Enabled Vehicular Networks Using Fog Computing

**DOI:** 10.3390/s23073543

**Published:** 2023-03-28

**Authors:** Zeyad Ghaleb Al-Mekhlafi, Mahmood A. Al-Shareeda, Selvakumar Manickam, Badiea Abdulkarem Mohammed, Abdulrahman Alreshidi, Meshari Alazmi, Jalawi Sulaiman Alshudukhi, Mohammad Alsaffar, Taha H. Rassem

**Affiliations:** 1 College of Computer Science and Engineering, University of Ha’il, Ha’il 81481, Saudi Arabia; 2 National Advanced IPv6 Centre (NAv6), Universiti Sains Malaysia, Gelugor 11800, Penang, Malaysia; 3 School of Computer Science and Informatics, De Montfort University, The Gateway, Leicester LE1 9BH, UK

**Keywords:** fog sever/computing, 5G, FC-CPPA, vehicular system, efficient authentication

## Abstract

Several researchers have proposed secure authentication techniques for addressing privacy and security concerns in the fifth-generation (5G)-enabled vehicle networks. To verify vehicles, however, these conditional privacy-preserving authentication (CPPA) systems required a roadside unit, an expensive component of vehicular networks. Moreover, these CPPA systems incur exceptionally high communication and processing costs. This study proposes a CPPA method based on fog computing (FC), as a solution for these issues in 5G-enabled vehicle networks. In our proposed FC-CPPA method, a fog server is used to establish a set of public anonymity identities and their corresponding signature keys, which are then preloaded into each authentic vehicle. We guarantee the security of the proposed FC-CPPA method in the context of a random oracle. Our solutions are not only compliant with confidentiality and security standards, but also resistant to a variety of threats. The communication costs of the proposal are only 84 bytes, while the computation costs are 0.0031, 2.0185 to sign and verify messages. Comparing our strategy to similar ones reveals that it saves time and money on communication and computing during the performance evaluation phase.

## 1. Introduction

The goal of the 5G-enabled vehicle network is to give users enhanced intelligence, security, and entertainment while on the road. This is because the transportation system includes the vehicle network [1,2,3,4]. Upon the arrival of the 5G era, all vehicles inside the vehicular network were required to have a wireless communication device installed, known as an onboard unit (OBU), for vehicle-to-everything (V2X) connectivity. Using the OBU gadget, the vehicles could send and receive messages with one another [5,6,7].

Since the 5G-enabled vehicular network’s communication is wireless and public, malicious actors may be able to alter or reply to, the communications exchanged between vehicles. The validity of the message can then be determined by the checker. Conditional privacy-preserving authentication (CPPA) systems were developed by a number of researchers as a means of satisfying both privacy and security concerns [6,8]. However, authenticating automobiles in these CPPA schemes necessitated the use of the roadside unit (RSU), which is an expensive part of vehicular networks. Furthermore, these CPPA systems incur extremely high communication and processing overhead [9]. Since fog computing reduces the amount of data transported to the cloud, it is the focus of this article rather than edge or cloud [10].

According to authors in [11], fog computing can meet the necessity of adopting vehicular networks by using a fog server instead of RSUs. It is considered that the fog server is not entirely trustworthy and has access to resources such as computing and storage. Meanwhile, based on authors in [12], fog computing has the potential to satisfy the need for implementing 5G networks. This study introduced a fog computing-based pseudonym authentication (FC-PA) strategy to lessen the performance burden in 5G-enabled automobile networks. The authors of [13] proposed a Chebyshev polynomial-based fog computing technique for 5G-enabled vehicle networks, which permits the revocation of pseudonyms.

Based on the related studies, several researchers introduced sophisticated privacy-authentication schemes to secure communication among vehicles. These schemes suffer from the massive overhead of the system in terms of computational and communicating costs. Meanwhile, the main question of this work is how our new architecture concept will reduce the system’s overhead. In the meantime, 5G-enabled vehicle networks based on fog computing solve a number of security and privacy problems. When dealing with these complications, real-time services in fog computing-based 5G-enabled vehicle networks require a complex CPPA architecture. As a result, we suggest a fresh FC-CPPA scheme architecture design for 5G-enabled vehicle networks that makes use of fog computing.

Here is how the rest of the paper is structured. The works that were referenced are discussed in Section 2. The preliminary information is detailed under Section 3. In Section 4, we propose fog-computing-based, 5G-enabled vehicle networks. The security element is then discussed in Section 5. The effectiveness of the proposed approach is detailed in Section 6. In Section 7, we conclude this paper.

## 2. Related Work

In this section, we review some of the related work. Recently, conditional privacy-preserving authentication (CPPA) schemes have played an important role in vehicular networks [14,15,16,17,18]. The following will highlight a few of the most recent relevant research.

For the vehicle network, Malhi and Batra [19] devised a bloom filter-based authentication system. Bloom filter technology can drastically cut down on the time and effort needed to verify a user’s signature.

Jiang et al. [20] offered a certificate revocation list-based anonymous batch authentication system (CRL). A CRL examination precedes signature verification and certificate issuance in automobile networks after a communication has been received. To combat privacy leaks and the requirement for numerous CRLs in terms of storage space, communication sources, and verification time, they proposed replacing the CRL check process with the computation of a hashed message authentication code.

Azees et al. [21] developed a practical method of message authentication for mobile networks. This technology can identify malevolent automobiles and block them from joining the network. Furthermore, this system can efficiently and anonymously validate automobiles via an RSU before broadcasting location-based service messages to nearby vehicles. However, in a practical setting, batch verification is not supported by this method.

Al-Shareeda et al. [22] suggested a secure CPPA method that is immune to impersonation attempts by ensuring that an adversary cannot use side-channel attacks to learn the true identity of the vehicle. There is no use of the map-to-point function in either the signing or verifying processes in this scheme.

Nonetheless, there are two major issues that affect the implementation of RSU-based CPPA schemes. One issue is that RSU makes some very strong security assumptions. Any RSU can render the entire authority system insecure if they gain access to the master key. The other major issue is the necessity for thousands of dollars to purchase RSUs. In addition, these methods employ the procedures of bilinear pairing cryptography (BPC). The bilinear pairing uses one curve of super-singular to construct scalar multiplication and pairing operations, which satisfies an 80-bit security requirement.

To cope with the challenge, Wu et al. [23] developed an ECC-based CPPA strategy for vehicular networks. A trusted authority (TA) is necessary for this method since it generates a set of anonymous public identifiers for each registered vehicle. In a later safe transfer, these identities and associated private keys are delivered to the public key generator.

Asaar et al. [24] introduced an elliptic curve-based proxy vehicle authentication system to prevent spoofing. By being resistant to forgeries and alterations in assaults against vehicle networks, this system also addresses problems with the preexisting approach.

Li et al. [25] developed a CPPA method for secure vehicle-to-vehicle communication that is both efficient and provably secure. In this method, during registration, the TA generates a set of public anonymity identities and preloads them with the appropriate signature keys into the car. However, this technique does not update these groups regularly, which can cause linkability problems.

Alshudukhi et al. [26] developed a CPPA technique for vehicular networks wherein vehicles can sign messages using a temporal key obtained from an RSU. Signature verification in this system is performed with the use of an elliptic curve.

Recently, several researchers [27,28,29,30,31,32,33] have invested a lot of interest topic in vehicular networks. They highlighted that the existing vehicular networks have several issues, including high movement speeds, no achievement of low delay demand, no support of scalability, and the existence of several security and privacy problems. To cope with these issues, 5G mobile communications are proposed. This is because 5G brings us lower latency and transmission delay. In addition, 5G offers flexible, customized services to users and supports line of sight (LOS) and device-to-device (D2D) communications which improve the efficiency performance of the system dramatically. Furthermore, some sensitive data can be stored or processed by fog computing to reduce cloud end burden and improve system efficacy. In light of this, 5G-enabled vehicle networks that incorporate the fog computing concept will be a major development in the near future of intelligent mobility.

Furthermore, we summarize the security comparison between our proposal and related work in Table 1. This paper proposes a CPPA scheme based on fog computing, namely, the FC-CPPA scheme for 5G-enabled vehicular networks. As we can view from Table 1, the schemes of [19,20,23] require the RSU component, while the schemes of [20,21,23] are vulnerable to achieving a mutual authentication. The scheme of Azees et al. [21] is vulnerable to resist replay attacks. Unlike the existing schemes, our work applies a lightweight operation based on ECC to propose an FC-CPPA scheme to sign the message and verify the signature.

## 3. Preliminaries

In this subsection, the system model design and D2D communication are presented separately.

### 3.1. System Model Design

**Figure 1:**
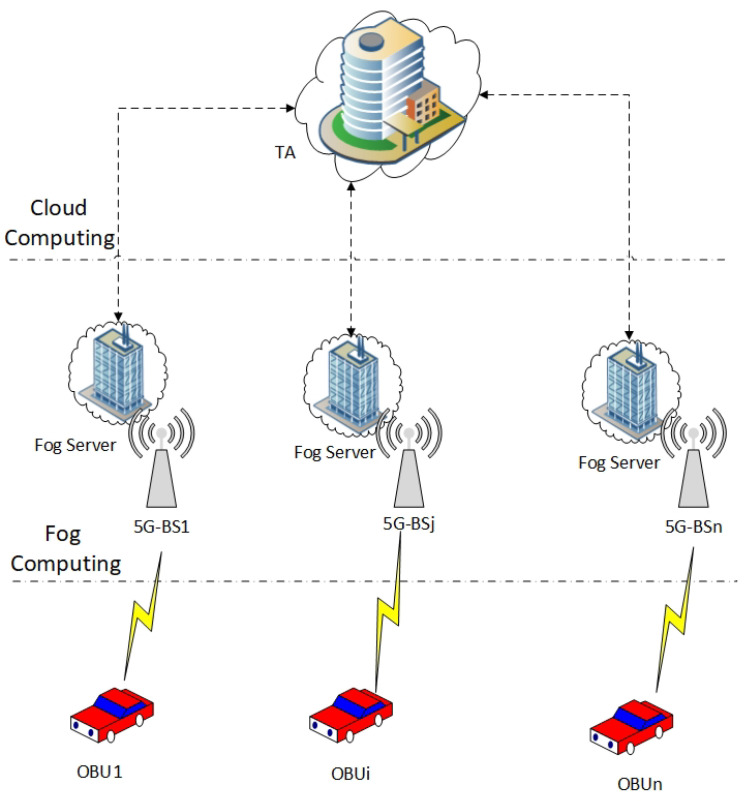
Our Proposed Architecture of the Fog Computing-based 5G-enabled Vehicular Networks.

According to novel research [11,34,35], we propose fog computing-based 5G-enabled vehicular networks, as shown in Figure 1. Our proposed architecture of the fog computing-based 5G-enabled vehicular networks consists of the following four components: one Trusted Authority (TA), some 5G-Base Stations (5G-BSs), some Fog Servers (FSs), and many vehicles.

- Trusted Authority (TA): It is assumed that the TA is a highly secure entity, which is reliable and independent. TA is responsible for initializing the system parameters for the 5G-enabled vehicular networks. Furthermore, it is in charge of registering each participating vehicle and fog server during the registration process.
- 5G-Base Stations (5G-BSs): The 5G-BSs are fixed infrastructure deployed on the roadside. It does not work with any computing and storage, only as an intermediate device between vehicles, fog servers, and TA. This is because it adapts to the wide range of D2D communication. Due to 5G-BSs being hardware, the attacker cannot compromise them.
- Fog Servers: This model assumes that the fog server has some verification computation and storage capabilities. The fog server has the private key of TA to validate vehicles during mutual authentication through 5G-BS. Furthermore, the fog server is responsible for issuing a group of public anonymous IDs and the corresponding group of signature keys to each participating vehicle.
- Vehicles: Each vehicle has a wireless device called an onboard unit (OBU) to communicate with other vehicles and fog servers. The OBU provides a tamper-proof device to save a group of public anonymous IDs and the corresponding group of signature keys that are obtained from the fog server. Meanwhile, the OBU offers a 5G protocol in order to exchange messages among 5G-BSs. When two mobile users can establish a connection directly, bypassing the base station (BS) and the core network, they are said to be engaging in device-to-device (D2D) transmission within the cellular network. Even if a node is within direct D2D communication range, all communications in a standard cellular network must first travel via the BS. With BS communication, traditional low data rate mobile services can thrive because users rarely contact directly with one another. However, users of today’s mobile networks make use of high-speed data services even when they are not in a direct line of sight to the network. This is why D2D communication has the potential to boost the spectral efficiency of networks. Spectrum efficiency, throughput, energy efficiency, latency, and fairness are all able to benefit from D2D communication [34,36].

### 3.2. Fog Computing

Our fog computing vehicles increase system computing power, minimize return pressure, and improve user service. The vehicle terminal can process the data instead of sending it to the remote core network TA. 5G-BS owns the fog computing’s data, therefore, the attacker cannot hack it like an RSU.

### 3.3. Design Goals

In order to ensure the safe environment of 5G-enabled vehicular networks, in this paper, the proposed FC-CPPA scheme should satisfy the following security and privacy requirements.

- Authentication and Integrity: Ensures that an attacker is not able to modify or forge messages sent from participating vehicles.
- Anonymity- and Privacy-Preserving: Ensures that an attacker is not able to disclose the identity of the participating vehicle.
- Unlinkability: Ensures that an attacker is not able to link two or more messages sent from the same participating vehicle.
- Traceability: Ensures that TA is able to trace the harmful vehicle.
- Revocability: Ensures that TA is able to revoke the harmful vehicle.
- Resist Security Attacks: Ensures that the proposed FC-CPPA scheme is able to resist security attacks such as forgery, modification, replay, and Man-in-The-Middle attacks.

## 4. The Proposed FC-CPPA Scheme

**Figure 2:**
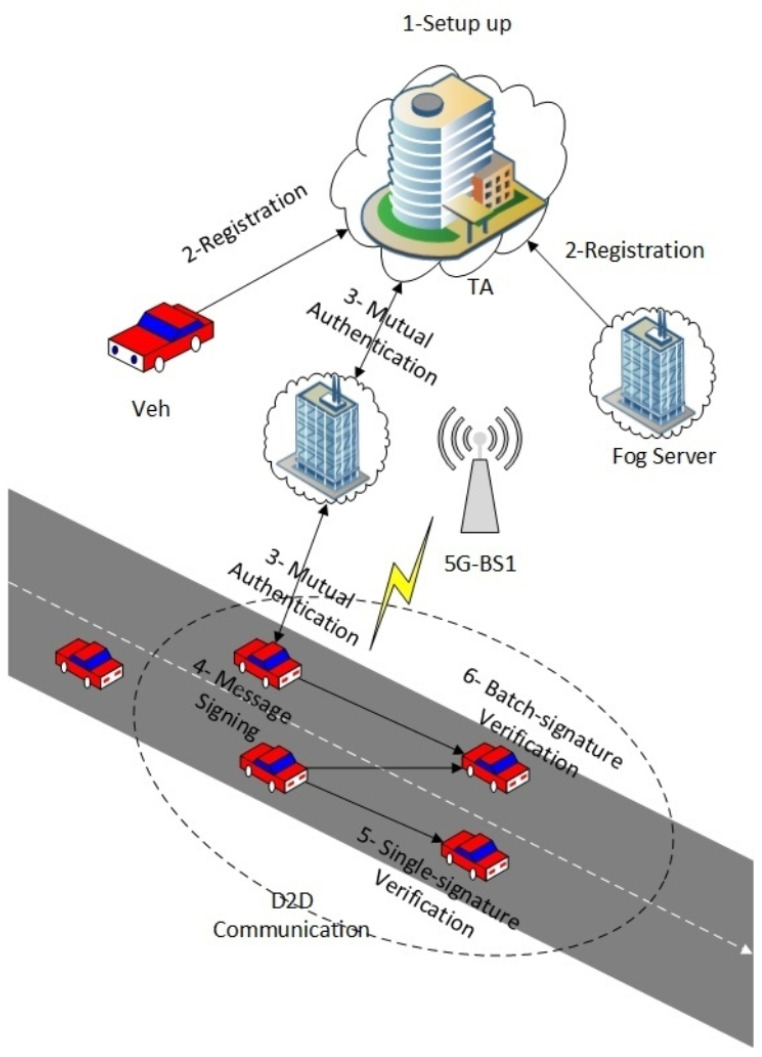
Overall Flow Chart of the Proposed FC-CPPA Scheme [14].

We present a fog computing-based conditional privacy-preserving authentication (FC-CPPA) technique for 5G-enabled vehicular networks, wherein the components can communicate. For obtaining crucial information through D2D mode, the proposed FC-CPPA method is of significant use. The proposed FC-CPPA technique consists of six stages: setup, registration, mutual authentication, message signing, single-signature verification, and batch-signature verification. The overall flow chart of the proposed FC-CPPA scheme is briefly presented below in Figure 2. The TA is responsible for issuing all nodes’ security parameters and hash functions. Then, TA registers the fog servers and vehicles by preloading the parameters during the registration phase. According to the mutual authentication phase, the vehicle requests the joining process to TA through fog computing to be considered a legal node for data sharing. Finally, the node signs and broadcasts shared messages; the verifier checks them once received during the message signing and signature verification phases.

### 4.1. Setup Phase

In this phase of the proposed FC-CPPA scheme, the TA executes the following steps.

- Let p,q be a large prime values, *G* be an additive group with the order *q* and a generator *P*, and *E* be a definition equation of an elliptic curve $y^{2}=x^{3}+ax+b$mod*p*, where $a,b\in Z_{q}^{*}$.
- TA sets the randomly chosen number $x\in Z_{q}^{*}$ as the private key and computes the relevant public key $Pub_{ta}=x\cdot P$.
- TA sets secure message authentication code (MAC) function MAC(·) and three the randomly chosen one-way hash functions $\left(h_{1},h_{2},h_{3}\right)$ as $h_{1}:G\to Z_{q}^{*}$$h_{2}:{\left\{0,1\right\}}^{*}\times {\left\{0,1\right\}}^{*}\times G\to Z_{q}^{*}$$h_{3}:{\left\{0,1\right\}}^{*}\to Z_{q}^{*}$.
- TA publishes the system parameters **SysPar =**$\left\{p,q,G,a,b,P,Pub_{ta},MAC\left(\cdot \right),h_{1},h_{2},h_{3}\right\}$.

### 4.2. Registration Phase

In this phase of the proposed FC-CPPA scheme, the TA is responsible for registering the fog server and the participating vehicle, which will be outlined separately in the following subsections.

#### 4.2.1. Fog Server Registration

The TA registers fog servers in the following steps.

- Fog server submits the identity $\left(ID_{fog_{j}}\right)$ to TA through secure channel.
- After checking the validity of $\left(ID_{fog_{j}}\right)$, TA computes $Pub_{fog_{j}}=x\cdot h_{1}\left(ID_{fog_{j}}\right)$ as the public key of the fog server.
- TA preloads the system parameters **SysPar =**$\{p,q,G,a,b,P,Pub_{fog_{j}},Pub_{ta},$$MAC\left(\cdot \right),h_{1},h_{2},h_{3}\}$ in each fog server.
- TA saves $\left(Pub_{fog_{j}}\right)$ into the fog registration list (FRL).
- TA saves the private key *x* on the TPD of the fog server through a secure channel.
- Finally, the fog server publishes a public key $Pub_{fog_{j}}$ through large-range communication of 5G-BSs.

#### 4.2.2. Vehicle Registration

The TA registers participating vehicles in the following steps.

- Vehicles submits the identity $\left(ID_{veh_{i}}\right)$ to TA through secure channel.
- After checking the validity of $\left(ID_{veh_{i}}\right)$, TA computes $IAID_{i}=x\cdot h_{1}\left(ID_{veh_{i}}\right)$ as inter anonymous-ID.
- TA saves the inter anonymous-ID $IAID_{i}$ on the TPD of the participating vehicle through a secure channel.
- TA saves $\left(IAID_{i},ID_{veh_{i}}\right)$ into vehicle registration list (VRL).
- Finally, TA preloads the system parameters **SysPar =**$\{p,q,G,a,b,P,Pub_{ta},$$MAC\left(\cdot \right),h_{1},h_{2},h_{3}\}$ in each OBU of participating vehicles.

### 4.3. Mutual Authentication Phase

In this phase of the proposed FC-CPPA scheme, prior to the vehicle $Veh_{i}$ broadcasting messages to others through D2D communication, it requires running a mutual authentication with the TA by helping fog server $Fog_{j}$. Using the large communication range of 5G-BSs, the details of mutual authentication are as the following steps.

- $Veh_{i}$: While receiving the public key $Pub_{fog_{j}}$ of fog server $Fog_{j}$, vehicle $Veh_{i}$ verifies whether Equation (1) holds or not. If (1) is so, vehicle $Veh_{i}$ continues the mutual authentication process. Otherwise, it is rejected.
  (1) $$Pub_{fog_{j}}\cdot P\overset{?}{=}h_{1}\left(ID_{fog_{j}}\right)\cdot Pub_{ta}$$
- $Veh_{i}\to Fog_{j}$: Vehicle $Veh_{i}$ sets the randomly chosen number $\alpha \in Z_{q}^{*}$ and computes its public anonymous-ID $PAID_{i}=\langle PAID_{i}^{1},PAID_{i}^{2}\rangle$, where $PAID_{i}^{1}=\alpha \cdot P$ and $PAID_{i}^{2}=IAID_{i}⊕h_{2}\left(\alpha \cdot Pub_{ta}\right)$. Next, vehicle $Veh_{i}$ sends $Msg_{veh_{i}\to fog_{j}}=\left\{PAID_{i},ts_{1},Sig_{veh_{i}\to fog_{j}}\right\}$ to fog server $Fog_{j}$, where $ts_{1}$ is the current timestamp and $Sig_{veh_{i}\to fog_{j}}=h_{3}(PAID_{i}^{1}||PAID_{i}^{2}||IAID_{i}||ts_{1})$ as the signature of $Msg_{veh_{i}\to fog_{j}}$.
- $Fog_{j}$: While receiving the $Msg_{veh_{i}\to fog_{j}}$ from vehicle $Veh_{i}$ through a 5G-BS, fog server $Fog_{j}$ initially verifies the freshness of timestamp $ts_{1}$ by checking whether Equation (2) holds or not for avoiding replay attacks. If (2) is verified, fog server $Fog_{j}$ continues the mutual authentication process. Otherwise, it is rejected.
  (2) $$ts_{i}>ts_{r}-ts_{▽}$$
  where $ts_{r}$ is the received time of $Msg_{veh_{i}\to fog_{j}}$ and $ts_{▽}$ is the predefined delay time.
- $Fog_{j}\to TA$: Next, fog server $Fog_{j}$ computes inter anonymous-ID of vehicle as $IAID_{i}=PAID_{i}^{2}⊕h_{2}\left(x\cdot PAID_{i}^{1}\right)$ and verifies the signature $Sig_{veh_{i}\to fog_{j}}$ of $Msg_{veh_{i}\to fog_{j}}$ by checking whether Equation (3) holds or not. If (3) is correct, the fog server $Fog_{j}$ continues the mutual authentication process. Otherwise, it is rejected.
  (3) $$Sig_{veh_{i}\to fog_{j}}^{-}\overset{?}{=}h_{3}(PAID_{i}^{1}||PAID_{i}^{2}||IAID_{i}||ts_{1})$$
  Then fog server $Fog_{j}$ sends $Msg_{fog_{j}\to ta}$ = $\{PAID_{i},ts_{1},Sig_{veh_{i}\to fog_{j}},Pub_{fog_{j}},ts_{2},$$Sig_{fog_{j}\to ta}\}$, where $Sig_{fog_{j}\to ta}$ = $h_{2}(PAID_{i}^{1}||PAID_{i}^{2}||IAID_{i}||ts_{1}||Pub_{fog_{j}}||ts_{2})$ as the signature of $Msg_{fog_{j}\to ta}$.
- $TA\to Fog_{j}$: While receiving the $Msg_{fog_{j}\to ta}$ from fog server $Fog_{j}$, TA checks the timestamp $ts_{2}$ and the signature $Sig_{fog_{j}\to ta}$ by using Equations (2) and (3), respectively. Then it computes Equation (4).
  (4) $$Sig_{fog_{j}\to ta}^{-}\overset{?}{=}h_{2}(PAID_{i}^{1}||PAID_{i}^{2}||IAID_{i}||ts_{1}||Pub_{fog_{j}}||ts_{2})$$
  Next, the TA checks the validity of participating vehicle and fog server by matching the existing value on VRL and FRL, respectively. If it is so, TA sends accept to fog server $Fog_{j}$. Otherwise, TA sends reject to fog server $Fog_{j}$.
- $Fog_{j}$: While receiving the $Msg_{ta\to fog_{j}}$ from TA, fog server $Fog_{j}$ picks n values $\beta_{l}\in Z_{q}^{*}$, where l=1:n. Then fog server $Fog_{j}$ computes a group of public anonymity-IDs $GPAID_{i}=\langle PAID_{il},\ldots ,PAID_{in}\rangle$ as follows, where l=1:n.
  (5) $$\begin{array}{cc} PAID_{in} & =\langle PAID_{il}^{1},PAID_{il}^{2}\rangle \\ \; & =\langle \beta_{l}\cdot P,IAID_{i}⊕h_{2}\left(\beta_{l}\cdot Pub_{ta}\right)\rangle \end{array}$$
  Then fog server $Fog_{j}$ computes the corresponding a group of signature keys $GSK_{i}=\langle SK_{il},\ldots ,SK_{in}\rangle$ based on the a group of public anonymity-IDs $GPAID_{i}$ as follows, where
  (6) $$\begin{array}{c} SK_{il}=x\cdot h_{2}(PAID_{il}^{1}||PAID_{il}^{2}) \end{array}$$
- $Fog_{j}$: Next, fog server $Fog_{j}$ sets the randomly chosen number $r\in Z_{q}^{*}$ and computes R=r·P and $A=r\cdot PAID_{i}^{1}=r\cdot \alpha \cdot P$. Then fog server $Fog_{j}$ computes $k_{ij}=h_{2}(A||IAID_{i})$ as a symmetric secret key between the vehicle $Veh_{i}$ and fog server $Fog_{j}$.
- $Fog_{j}\to Veh_{i}$: Next, fog server $Fog_{j}$ encrypts $EncAuth_{fog_{j}}=MAC_{k_{ij}}(\beta_{l}||GPAID_{i}||GSK_{i})$ and sends $Msg_{fog_{j}\to veh_{i}}$ = $\left\{EncAuth_{fog_{j}},ts_{3},R,Sig_{fog_{j}\to veh_{i}}\right\}$, where $Sig_{fog_{j}\to veh_{i}}$ = $h_{2}(\beta_{l}||GPAID_{i}||GSK_{i}||ts_{3}||R||IAID_{i})$.
- $Veh_{i}$: While receiving the $Msg_{fog_{j}\to veh_{i}}$ from fog server $Fog_{j}$, vehicle $Veh_{i}$ verifies the freshness of timestamp $ts_{3}$ by using Equation (2). Then vehicle $Veh_{i}$ computes α·R, $k_{ij}=h_{2}(A||IAID_{i})$ and decrypts $DecAuth_{Veh_{i}}=MAC_{k_{ij}}\left(EncAuth_{fog_{j}}\right)$ to obtain $\left(\beta_{l},GPAID_{i},GSK_{i}\right)$.
- $Veh_{i}$: Next, vehicle $Veh_{i}$ verifies the $Msg_{fog_{j}\to veh_{i}}$ by checking whether Equation (7) holds or not.
  (7) $$Sig_{fog_{j}\to veh_{i}}^{-}\overset{?}{=}h_{2}(\beta_{l}||GPAID_{i}||GSK_{i}||ts_{3}||R||IAID_{i})$$

Note that the vehicle $Veh_{i}$ has a group of n public anonymity-IDs $GPAID_{i}$ to sign messages in the large communication range of 5G-BSs through D2D communication.

### 4.4. Message Signing Phase

Vehicle $Veh_{i}$ creates a digital signature of safety-related message $SMsg_{i}$ as part of the proposed FC-CPPA scheme. Once the anonymous-ID, timestamp, message, and digital signature have been broadcast, additional cars within the 5G-BS coverage region will be able to receive them from the vehicle $Veh_{i}$. In the following steps, we will demonstrate how to sign a message.

- Vehicle $Veh_{i}$ picks unused public anonymous-ID $PAID_{i}$ and the corresponding signature key $SK_{i}$ from group of $GPAID_{i}$ and $GSK_{i}$, receptively.
- Vehicle $Veh_{i}$ signs messages as follows.
  (8) $$\sigma_{Veh_{i}}=\beta_{i}\cdot h_{3}(SMsg_{i}||PAID_{i}^{1}||PAID_{i}^{2}||ts_{i})+SK_{i}$$
- Finally, vehicle $veh_{i}$ broadcasts $Msg_{veh_{i}}=$$\left(SMsg_{i},PAID_{i}^{1},PAID_{i}^{2},ts_{i},\sigma_{Veh_{i}}\right)$ to others within the communication range of 5G-BS.

### 4.5. Single-Signature Verification Phase

In this phase of the proposed FC-CPPA scheme, prior to accepting safety-related message $SMsg_{i}$ of $Msg_{veh_{i}}=$$\left(SMsg_{i},PAID_{i}^{1},PAID_{i}^{2},ts_{i},\sigma_{Veh_{i}}\right)$ from vehicle $veh_{i}$, the verifier $Ver_{i}$ should check the freshness of timestamp $ts_{i}$ and the validity of signature $\sigma_{Veh_{i}}$ by verifying whether both Equations (2) and (9) hold or not.

(9) $$\begin{array}{cc} \; & \sigma_{Veh_{i}}\cdot P\overset{?}{=}\left(\beta_{i}\cdot h_{3}(SMsg_{i}||PAID_{i}^{1}||PAID_{i}^{2}||ts_{i})+SK_{i}\right)\cdot P\\ \; & \overset{?}{=}\left(\beta_{i}\cdot h_{3}(SMsg_{i}||PAID_{i}^{1}||PAID_{i}^{2}||ts_{i})+x\cdot h_{2}(PAID_{i}^{1}||PAID_{i}^{2})\right)\cdot P\\ \; & \overset{?}{=}h_{3}(SMsg_{i}||PAID_{i}^{1}||PAID_{i}^{2}||ts_{i})\cdot \beta_{i}\cdot P+h_{2}(PAID_{i}^{1}||PAID_{i}^{2})\cdot x\cdot P\\ \; & \overset{?}{=}h_{3}(SMsg_{i}||PAID_{i}^{1}||PAID_{i}^{2}||ts_{i})\cdot PAID_{i}^{1}+h_{2}(PAID_{i}^{1}||PAID_{i}^{2})\cdot Pub_{ta} \end{array}$$

### 4.6. Batch-Signature Verification Phase

While receiving n of $Msg_{veh_{i}}=$$\left({SMsg_{i}}^{1},{PAID_{i}^{1}}^{1},{PAID_{i}^{2}}^{1},{ts_{i}}^{1},{\sigma_{Veh_{i}}}^{1}\right)$,…, $({SMsg_{i}}^{n},$${PAID_{i}^{1}}^{n},{PAID_{i}^{2}}^{n},{ts_{i}}^{n},{\sigma_{Veh_{i}}}^{n})$ from n vehicles, the verifier $Ver_{i}$ should be checked the freshness of n timestamps ${ts_{i}}^{n}$ and the validity of n signatures ${\sigma_{Veh_{i}}}^{n}$ simultaneously. the verifier $Ver_{i}$ uses $\gamma =\{\gamma_{1},\gamma_{2},\ldots ,\gamma_{n}\}$ as small the small exponent test technology [37,38] to achieve non-reputation in the batch signature verification. Therefore, the verifier $Ver_{i}$ should check the freshness of n timestamp ${ts_{i}}^{n}$ and the validity of n signature ${\sigma_{Veh_{i}}}^{n}$ by verifying whether both Equations (2) and (10) hold or not.

(10) $$\begin{array}{cc} \; & \left(\sum_{i=1}^{n}\gamma_{i}\cdot \sigma_{Veh_{i}}\right)\cdot P\overset{?}{=}\sum_{i=1}^{n}\gamma_{i}\cdot \left(\beta_{i}\cdot h_{3}(SMsg_{i}||PAID_{i}^{1}||PAID_{i}^{2}||ts_{i})+SK_{i}\right)\cdot P\\ \; & \overset{?}{=}\sum_{i=1}^{n}\gamma_{i}\cdot \left(\beta_{i}\cdot h_{3}(SMsg_{i}||PAID_{i}^{1}||PAID_{i}^{2}||ts_{i})+x\cdot h_{2}(PAID_{i}^{1}||PAID_{i}^{2})\right)\cdot P\\ \; & \overset{?}{=}\sum_{i=1}^{n}\gamma_{i}\cdot \left(h_{3}(SMsg_{i}||PAID_{i}^{1}||PAID_{i}^{2}||ts_{i})\cdot \beta_{i}\cdot P+h_{2}(PAID_{i}^{1}||PAID_{i}^{2})\cdot x\cdot P\right)\\ \; & \overset{?}{=}\sum_{i=1}^{n}\gamma_{i}\cdot h_{3}(SMsg_{i}||PAID_{i}^{1}||PAID_{i}^{2}||ts_{i})\cdot PAID_{i}^{1}+\left(\sum_{i=1}^{n}\gamma_{i}\cdot h_{2}(PAID_{i}^{1}||PAID_{i}^{2})\right)\cdot Pub_{ta} \end{array}$$

## 5. Security Analysis

This section explains the security analysis in terms of formal analysis and security requirements.

### 5.1. Formal Analysis

**Figure 3:**
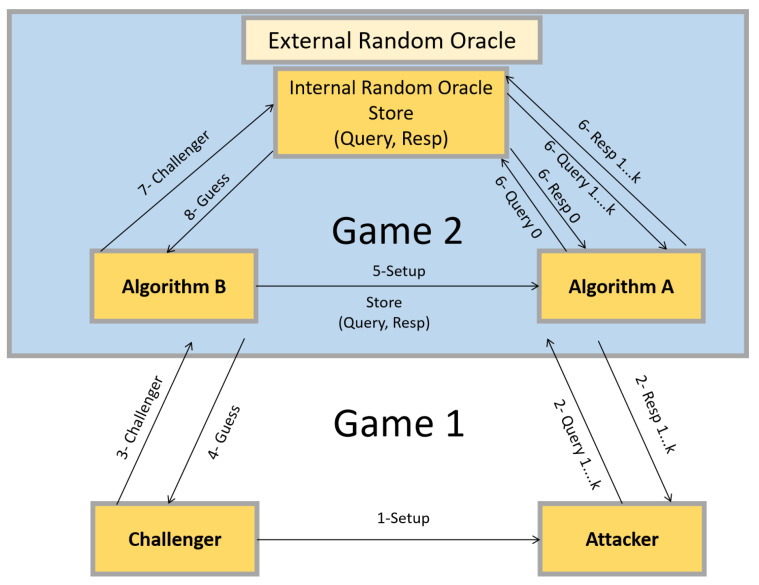
Steps of Random Oracle Model.

We propose several games that an adversary could use to attack the proposed FC-CPPA method for vehicular networks’ message integrity, authentication, and identity privacy. Figure 3 shows the steps of the random oracle model.

#### 5.1.1. Authentication and Integrity

In this paper, we show that in the proposed FC-CPPA scheme, an attacker cannot produce a valid signature on behalf of any vehicle. Forgery-proof communication between vehicles relies on the hardness of the computational Diffie–Hellman (CDH) issue. Our solution is secure against adaptive selected message attacks and existential forgery when utilizing the random oracle paradigm. The following is our evidence: For all messages sent by vehicles, the signature $\sigma_{Veh_{i}}$ is computed on the message $SMsg_{i}$ by using the $SK_{il}$ key, where $SK_{il}=x\cdot h_{2}(PAID_{il}^{1}||PAID_{il}^{2})$. Considering an adversary, the fog server has only accessibility to the number of the system’s master key *x*, and also the external adversary has only accessibility to the public key $Pub_{ta}=x\cdot P$. Consequently, the adversary cannot impersonate the signature based on the hardness of the decision Diffie-Hellman (DDH) problem. First, let us suppose that a vehicle serves as both the challenger and the attacker in **Game 1**:

**Setup:** The challenger sends the adversary the parameters of system *P*, $Pub_{ta}$, and $PAID_{in}$$=\langle PAID_{il}^{1},PAID_{il}^{2}\rangle$. The adversary can eavesdrop $PAID_{in}$ for each vehicle having $OBU_{i}$ in this situation, which is comparable to the situation when they can do so from the air.

**Query:** Suppose the adversary is unable to compute the functions of $h_{1}\left(\cdot \right)$, $h_{2}\left(\cdot \right)$, and $h_{3}\left(\cdot \right)$. The adversary can only demand the amount $h_{2}(PAID_{il}^{1}||PAID_{il}^{2})$ and the hash function $h_{3}\left(\cdot \right)$ up to n various messages from the challenger.

**Challenge:** In order to generate $\sigma_{Veh_{i}}$ and $\sigma_{Veh_{j}}$, the challenger requires the adversary to pick two arbitrary messages ($SMsg_{i}$ and $SMsg_{j}$) and sign them on behalf of $V_{i}$.

**Guess:** Two pairs ($SMsg_{i},\sigma_{Veh_{i}}$) and ($SMsg_{j},\sigma_{Veh_{j}}$) are sent to the challenger by the adversary.

Pr[$\sigma_{Veh_{i}}$ and $\sigma_{Veh_{j}}$ are legitimate signatures] is determined as the advantage of the adversary. Our work is resistant to existential forgery and adaptive chosen message attacks through a random oracle model if the advantage of the adversary is negligible.

**Next:** The algorithm *A*, which we will call the attacker in **Game 1**, is polynomially fast and has a significant performance advantage over *e*. We next construct **Game 2**, where an attacker in the Diffie–Hellman problem (DHP) can utilize *A* to gain a significant advantage.

The amounts **SysPar** = $\left\{p,q,G,a,b,P,Pub_{fog_{j}},Pub_{ta},MAC\left(\cdot \right),h_{1},h_{2},h_{3}\right\}$ are taking *B* as inputs, and then *B* is requested to compute a(2c+bd)P. This calculation is as hard as the calculation of acP.

We now go into more detail about how *B* can use *A* to resolve the DHP in the manner described below:

**Setup:***B* selects the elements (*P*, $Pub_{ta}=x\cdot P$) in this paper, where *x* has a role of the private key. *B* offers a randomly inter anonymous-ID $IAID_{i}$ and a public anonymity-IDs $PAID_{in}$ = $\langle PAID_{il}^{1},PAID_{il}^{2}$〉=$\langle \beta_{l}\cdot P,IAID_{i}⊕h_{2}\left(\beta_{l}\cdot Pub_{ta}\right)\rangle$ for *A*.

Note that *A* is a vehicle and understands how to verify the combination of public anonymity IDs. Therefore, $IAID_{i}$ must be properly formulated such that *A* cannot be in any question about it.

**Query:** Initially, *A* requests that *B* pay the sum of $h_{2}(PAID_{il}^{1}||PAID_{il}^{2})$, and then *B* gives *A* the sum of bP as a response. The second step is for *A* to pick *K* different messages at random and have *B* provide the hash value of those messages. Finally, *B* uses a random oracle to respond to these requests and keeps track of the results in a database table. *B* will react to a message with a predetermined amount if the sender has been asked for the same information in the past. Unless a *u* or *v* is requested, *B* will return a random number and save it to a table. If *r* is a random number, then *B* will respond to the $u^{th}$ (relating to the message SMsgu) and $v^{th}$ (relating to the message $SMsg_{u}$) questions with *r* and d−r, respectively, (*d* > *r*).

**Challenge:***B* answers *A* to randomly select two messages $SMsg_{i}$ and $SMsg_{j}$, and then make a signature on them rather than $V_{i}$.

**Guess:** Two messages $SMsg_{i}$ and $SMsg_{j}$ are randomly chosen by *A*, and then it computes the signatures $\sigma_{Veh_{i}}$ and $\sigma_{Veh_{j}}$ rather than $V_{i}$. Notice that when *A* required $SMsg_{i}$ and $SMsg_{j}$ before, *A* understands that how to compute $h_{3}\left(SMsg_{i}\right)$ and $h_{3}\left(SMsg_{j}\right)$. *B* computes $\sigma_{Veh_{i}}+\sigma_{Veh_{j}}$ when $SMsg_{i}=SMsg_{u}$ and (or vice versa, i.e., $SMsg_{i}=SMsg_{v}$ and $SMsg_{j}=SMsg_{u}$). This case equals $\sigma_{Veh_{i}}+\sigma_{Veh_{j}}=\beta_{i}\cdot h_{3}(SMsg_{i}||PAID_{i}^{1}||PAID_{i}^{2}||ts_{i})+x\cdot h_{2}(PAID_{il}^{1}||PAID_{il}^{2})+\beta_{i}\cdot h_{3}(SMsg_{j}||PAID_{i}^{1}||PAID_{i}^{2}||ts_{i})+x\cdot h_{2}(PAID_{il}^{1}||PAID_{il}^{2})$.

*B* successfully resolves the DHP owing to *B* having the amount of the past equation. Let *e* be the advantage of adversary *A* in breaking the our scheme and $\frac{1}{C(k,2)}$ be the probability which *A* selects $SMsg_{u}$ and $SMsg_{v}$. Therefore, the success probability of resolving the taking DHP example is Pr[*B* succeeds] = $\frac{1}{C(k,2)}\cdot e$.

Assuming *e* is non-negligible, *B* can use the success probability to find a solution to the DHP. Considering the widespread belief that DHP is challenging, this result is surprising. Given the nature of the random oracle model, our system is immune to adaptive selected message assaults and existential forgery.

#### 5.1.2. Identity Privacy-Preserving

The only data entered into the network that is pertinent to the vehicle’s true identity is its public anonymity identity. We explain why, even if the adversary has accessibility to the vehicle’s public anonymity identity, they cannot simply discover the true identity of the vehicle in the paragraphs that follow. We describe how, if DDHP is challenging, the vehicle’s public anonymity identity can safeguard the true identity of the vehicle. Our evidence is as follows:

First, let us suppose that the challenger and adversary are in the **Game 1**, as shown below.

**Setup:** The parameters of the system are given to the opponent by the challenger, who is represented by {*P*, $Pub_{ta}$}.

**Select:** To the challenger, the opponent selects two true identities, $IAID_{0}$ and $IAID_{1}$. Note that the selections made do not need to be random.

**Challenge:** The adversary has zeroed out bit *r* with a probability of $\frac{1}{2}$. After that, the challenger will relay the attacker’s genuine identity, $IAID_{r}$.

**Guess:** To gain an advantage, the adversary guesses the bit *r* selected by the challenger and receives the guess as $r^{-}\cdot Pr\left[r=r^{-}\right]-\frac{1}{2}$. We prove that our public anonymity identity generation is secure against a selected plaintext attack, provided that the adversary’s advantage is small.

Next, we consider the **Game 1** opponent as a polynomial-time algorithm *A* with a non-trivial advantage *e*. Next, we build **Game 2**, in which the adversary in the DDHP issue has a non-negligible advantage thanks to *A*.

A DDHP example (*P*, aP, bP, *T*) is taken to *B* as input, and then *B* is demanded to assign whether T=abP or not. Let *t* be a bit guessed by *B*, i.e., t = 0 for positive replay (T = abP) and t = 1 for negative replay (T ≠ abP). Now, we explain how *B* can use *A* to resolve the DDHP problem in the manner described below:

**Setup:** Based on the DDHP example, *B* chooses and sends *A* the parameters (P, aP, bP, T), with *a* playing the role of the master key *x*. **Choose:** For the purpose of verification, *A* selects two public keys ($IAID_{0}$ and $IAID_{1}$) and sends them to *B*. **Challenge:** The adversary has zeroed bit *r* with a probability of $\frac{1}{2}$. Next, the challenger will provide the opponent with the anonymous identification $IAID_{r}$.

**Guess:** The adversary makes use of the challenger to make a guess at the bit *r*, and the adversary’s advantage is calculated to be $r^{-}\cdot Pr\left[r=r^{-}\right]-\frac{1}{2}$. Our public anonymity identity generation is secure against a selected plaintext attack if the disadvantage to the adversary is small enough (CPA). Next, we think of the **Game 1** opponent as a polynomial-time algorithm *A* that has a non-trivial advantage *e*. Next, we build **Game 2**, in which the adversary in the DDHP issue has a non-negligible advantage thanks to *A*. We provide *B* an example of DDHP (*P*, aP, bP, *T*) and require *B* to decide if T=abP. Let *t* stand for the *B*-guessed bit: t = 0 for successful replay (T = abP) and t = 1 for unsuccessful replay (T neq abP). The following describes how *B* can implement *A* to solve the DDHP problem.

**Setup:** Using the DDHP example as a guide, *B* chooses and sends *A* the parameters (P, aP, bP, T), with *a* playing the role of the master key *x*.

**Choose:** In this scenario, *A* selects two verification public keys, $IAID_{0}$ and $IAID_{1}$, and sends them to *B*.

**Challenge:** Now *B* has the key of the challenger, so *B* can arbitrarily set a bit *r* and generates the public anonymity-IDs $PAID_{in}$ = $\langle PAID_{il}^{1},PAID_{il}^{2}\rangle$, in which $\langle PAID_{il}^{1}=\beta_{l}\cdot P,PAID_{il}^{2}=IAID_{i}⊕h_{2}\left(\beta_{l}\cdot Pub_{ta}\right)\rangle$ and $\beta_{l}$ is a random nonce, and transmits it to *A*.

**Guess:** Finally, *A* will send *B* a bit representing their best estimate of *r*, denoted by $r^{-}$. *B* gives the right answer to the DDHP issue if and only if the guess is correct. How *B* solves the DDHP problem is what we are focusing on right now. This is a good form of the equation: $IAID_{r}⊕h_{2}\left(\beta_{l}bP\right)=h_{2}\left(bPAID_{il}^{1}\right)$ if and only if T = $\beta_{l}$ bP (t = o). Due to *A*’s non-negligible advantage in the aforementioned game, *A* may violate the suggested FC-CPPA technique and estimate *x* with probability $\frac{1}{2}+e$. Therefore, at time t = 0, there is a $\frac{1}{2}+e$ probability that *B* will successfully transmit (i.e., Pr[*B* succeeds| t = 0] = $\frac{1}{2}+e$). The word H(zT) cannot be eliminated by the term H(bIAID1) when *T* is selected at random. As a result, the computation reveals nothing about *r*, denoted by Pr[Bsucceeds|t=1]=frac12. With *r* hidden, *A* can only guess its value with the probability Pr[*B* succeeds] = $\frac{1}{2}\cdot \left(\frac{1}{2}+e\right)+\frac{1}{2}\cdot \frac{1}{2}=\frac{1}{2}+\frac{e}{2}$. Since *e* is small, *B* can solve the DDHP problem. This argument counters the view that DDHP is difficult. In this way, the proposed FC-CPPA method can protect individuals’ identities while still allowing for the public anonymity of automobiles.

Furthermore, the random nonce $\beta_{l}$ ensures that each public anonymity ID is unique. Because of this, following the vehicle’s movements is impossible unless one has access to the shared key $k_{ij}$ between the fog server and the automobile.

### 5.2. Informal Analysis

Our suggested FC-CPPA scheme for 5G-enabled vehicular networks should adhere to the highest standards of security and privacy, as discussed below.

- Authentication and Integrity: The format of the message shared by vehicle is $Msg_{veh_{i}}=$$\left(SMsg_{i},PAID_{i}^{1},PAID_{i}^{2},ts_{i},\sigma_{Veh_{i}}\right)$ to other vehicles, where $\sigma_{Veh_{i}}=\beta_{i}\cdot h_{3}(SMsg_{i}\left|\right|$$PAID_{i}^{1}||PAID_{i}^{2}||ts_{i})+SK_{i}$ is the signature message. Before message $SMsg_{i}$ is accepted, the checker computes Equations (9) and (10) to detect any modification/impersonation of the message. Hence, the authentication and integrity requirements are achieved in our work.
- Anonymity- and Privacy-Preserving: During the vehicle registration phase, once the vehicle submits its identity $\left(ID_{veh_{i}}\right)$ to TA through a secure channel, the TA computes and preloads inter anonymous-ID $IAID_{i}=x\cdot h_{1}\left(ID_{veh_{i}}\right)$ to the vehicle. By using inter anonymous-ID $IAID_{i}$, the fog server computes and preloads a group of public anonymous-ID $GPAID_{i}$ to the vehicle during the mutual authentication process. The vehicle picks unused public anonymous-ID $PAID_{i}$ from group of $GPAID_{i}$ that are received from the fog server, where $PAID_{i}=\langle PAID_{i}^{1},PAID_{i}^{2}\rangle =\langle \beta_{l}\cdot P,IAID_{i}⊕h_{2}\left(\beta_{l}\cdot Pub_{ta}\right)\rangle$ at a time. Since the random numbers $\beta_{l}$ and *x* are not known by the attacker, it is possible to reveal the true identity of the vehicle. Hence, the anonymity privacy-preserving requirement is achieved in our work.
- Unlikability: When the vehicle wants to broadcast a message, it picks unused public anonymous-ID $PAID_{i}$ and the corresponding signature key $SK_{i}$ from the group of $GPAID_{i}$ and $GSK_{i}$ that is received from fog server, receptively, where $PAID_{i}=\langle PAID_{i}^{1},PAID_{i}^{2}\rangle =\langle \beta_{l}\cdot P,IAID_{i}⊕h_{2}\left(\beta_{l}\cdot Pub_{ta}\right)\rangle$. Since the random nonce $\beta_{l}$ is included for each public anonymous-ID $PAID_{i}$, the attacker does not have the ability to link several messages sent from the same source. Hence, the unlikability requirement is achieved in our work.
- Traceability: The TA and the fog server work together to locate and revoke the harmful vehicle after receiving a report about a harmful vehicle. The TA discovers the vehicle’s inter anonymous-ID as follows:
  (11) $$IAID_{i}=PAID_{il}^{2}⊕h_{2}\left(x\cdot PAID_{il}^{1}\right)$$
  Hence, the traceability requirement is achieved in our work.
- Revocability: Once the traceability requirement is complete, the TA then updates the CRL by adding the inter anonymous-ID $IAID_{i}$, and sends the new CRL to fog servers. As a result, the local CRLs are updated and broadcast by the fog servers involving the victim’s vehicle. Additionally, the authentication procedure fails in line with Equation (8) when the malicious actor joins the new fog server region, or the valid timestamp $ts_{i}$ has expired. Hence, the revocability requirement is achieved in our work.

### 5.3. Attack Scenarios

In this subsection, proof of security attack resistance on the proposed FC-CPPA scheme is provided. This paper focuses on general security attacks such as replay, modify, forgery, and MITM attacks. These attacks try to damage the system and create accidents among vehicles in the road environment. The following steps are provided to show how our proposal FC-CPPA scheme is resistant to these attacks.

- Resistance to Replay Attacks: The timestamp $ts_{i}$ is included the message format $Msg_{veh_{i}}=$$\left(SMsg_{i},PAID_{i}^{1},PAID_{i}^{2},ts_{i},\sigma_{Veh_{i}}\right)$ in our proposal. Before the message $SMsg_{i}$ is accepted, the checker verifies the freshness of timestamp $ts_{i}$ by checking whether Equation (12) holds or not to avoid replay attacks. If (12) is verified, the checker continues the mutual authentication process. Otherwise, it is rejected.
  (12) $$ts_{i}>ts_{r}-ts_{▽}$$
  where $ts_{r}$ is the received time of $\left(SMsg_{i},PAID_{i}^{1},PAID_{i}^{2},ts_{i},\sigma_{Veh_{i}}\right)$ and $ts_{▽}$ is the predefined delay time. Hence, replay attacks are resisted in our work.
- Resistance to Modify Attacks: The attacker cannot modify the message $SMsg_{i}$ from $Msg_{veh_{i}}=$$\left(SMsg_{i},PAID_{i}^{1},PAID_{i}^{2},ts_{i},\sigma_{Veh_{i}}\right)$ sent by a vehicle. This is because the checker computes Equations (9) and (10) to detect any message modification. Hence, modification attacks are resisted in our work.
- Resistance to Forgery Attacks: The adversary cannot impersonate the true identity of the vehicle from $Msg_{veh_{i}}=$$\left(SMsg_{i},PAID_{i}^{1},PAID_{i}^{2},ts_{i},\sigma_{Veh_{i}}\right)$ sent by vehicle. This is because the checker computes Equations (9) and (10) to detect any impersonation of the message. Hence, forgery attacks are resisted in our work.
- Resistance to Man-In-The-Middle Attacks: According to the above analysis, no attacker is able to change/modify/replay/impersonate the communication between the sender and receiver. Hence, man-in-the-middle attacks are resisted in our work.

## 6. Performance Evaluation and Comparison

We evaluate and compare our FC-CPPA scheme with schemes of Malhi and Batra [19], Jiang et al. [20], Azees et al. [21] and Wu et al. [23] with regard to the costs of communication and computation. Since the time required to perform a general cryptographic hash function has a very small value in processing, it has been excluded from this article. In order to ensure that pseudonym authentication systems may meet the 80-bit security threshold, this study chooses bilinear pairings $e^{-}:G_{1}$×$G_{1}\to G_{2}$. In this case, $G_{2}$ and $G_{1}$ each represent a 160-bit prime order multiplicative group and a cyclic additive group, respectively, with generator *P*. The point *P* has a prime size of 512 bits and is based on the supersingular curve $y^{2}\equiv \left(x^{3}+x\right)modp$ of embedded degree 2.

### 6.1. Experimental Environment

In this paper, the experimental environment used is described. Table 2 summarizes the employed software and hardware specifications. This experiment is based on the MIRACL library [39] to execute and run the cryptography operations of bilinear pair and elliptic curve. Hence, the sum was calculated using the elapsed time of each individual process for operations. The overhead expense is equal to the Elapsed Time (ET) between the exit and the entry to each phase as Equation (13).

(13) $$ET=T_{i}^{out}-T_{i}^{in}$$

where $T_{i}^{out}$ is the exit times of completed operations and $T_{i}^{in}$ is the entrance times of completed operations. By utilizing the MIRACL library in this paper, we can see the basic cryptographic operation and its running time in Table 3.

### 6.2. Computation Cost

At each stage of the process—signing messages, verifying individual signatures, and verifying a batch of signatures—we examine and compare the computational costs of our work to those of similar methods.

The scheme of Azees et al. [21] executes $4M_{bp}\approx 2.6872$ ms, $2P_{bp}+5M_{bp}+2A_{bp}\approx 19.4702$ ms, and $\left(n+1\right)P_{bp}+5nM_{bp}+2nA_{bp}\approx 13.6592n+5.811$ ms for MsgSign phase, SigVerify phase, and BSigVerify phase, respectively. The scheme of Al-Shareeda et al. [22] executes $1M_{bp}\approx 1.5654$ ms, $2P_{bp}+2M_{bp}+1A_{bp}\approx 6.2722$ ms, and $P_{bp}+2nM_{bp}+nA_{bp}\approx 3.1414n+5.811$ ms for MsgSign phase, SigVerify phase, and BSigVerify phase, respectively. The scheme of Asaar et al. [24] executes $7M_{ecc}\approx 4.7026$ ms, $12M_{ecc}+8A_{ecc}\approx 8.0864$ ms, and $\left(4n+10\right)M_{ecc}+\left(6n+2\right)A_{ecc}\approx 2.7058n+6.7242$ ms for MsgSign phase, SigVerify phase, and BSigVerify phase, respectively. The scheme of Li et al. [25] executes $1M_{ecc}\approx 0.6718$ ms, $4M_{ecc}+1A_{ecc}\approx 2.6903$ ms, and $\left(2n+2\right)M_{ecc}+nA_{ecc}\approx 1.3467n+1.3436$ ms for MsgSign phase, SigVerify phase, and BSigVerify phase, respectively. The scheme of Alshudukhi et al. [26] executes $2M_{ecc}+1A_{ecc}\approx 1.3467$ ms, $3M_{ecc}+1A_{ecc}\approx 2.0185$ ms, and $\left(n+2\right)M_{ecc}+\left(n-1\right)A_{ecc}\approx 0.6749n+1.3405$ ms for MsgSign phase, SigVerify phase, and BSigVerify phase, respectively. The scheme of our proposal for FC-CPPA executes $1A_{ecc}\approx 0.0031$ ms, $3M_{ecc}+1A_{ecc}\approx 2.0185$ ms, and $\left(n+2\right)M_{ecc}+\left(n-1\right)A_{ecc}\approx 0.6749n+1.3405$ ms.

From Table 4, it clearly shows that our FC-CPPA scheme has the computation cost advantage over the related work at the message signing phase because our FC-CPPA scheme takes only 0.0031 ms whereas the related schemes of Azees et al. [21], Al-Shareeda et al. [22], Asaar et al. [24], Li et al. [25], and Alshudukhi et al. [26] take 2.6872 ms, 1.5654 ms, 4.7026 ms, 0.6718 ms, and 1.3467 ms, respectively. To verify a single signature, our FC-CPPA scheme requires only 2.0185, while to verify the batch signature, our work needs 0.6749n+1.3405. Therefore, the calculation costs of the message signing phase, the individual signature verification stage, and the group signature verification stage in our FC-CPPA scheme are less than those of the existing algorithms, even when the traffic load grows.

### 6.3. Communication Cost

The size of a single point in $G_{1}$, $G_{1}^{-}$, $G_{2}^{-}$ is 128 bytes, while the size of a single point in *G* is 40 bytes, all based on the parameters established in the preceding section. The output of a timestamp is 4 bytes, while that of a hash function is 20 bytes. Table 5 details the costs associated with the various forms of communication used in our proposed and related work.

From Table 5, we are able to deduce that our approach has a lower communication cost than current techniques.

## 7. Conclusions

In this study, we present a novel conditional privacy-preserving authentication strategy for 5G-enabled vehicle networks using fog computing. The proposed FC-CPPA design does not necessitate the usage of RSUs for purposes of verification, storage, or computation. In the broad area serviced by 5G-BSs, the fog server provides and preloads a set of public anonymity-IDs and the matching signature key into each participating vehicle. The security study shows that our idea is safe from adaptive selected message attacks and existential forgeries when operating in a random oracle paradigm. While doing so, our work meets the criteria for authentication and integrity, maintaining anonymity and privacy, traceability, revocability, unlinkability, resistance to replay, resistance to forging, resistance to modification, and resistance to man-in-the-middle attacks. The part devoted to evaluating and contrasting performance demonstrates, finally, that our FC-CPPA scheme performs better than other studies in terms of communication and computational costs.

In future work, we address the performance evaluation of this solution by proposing a chaos map algorithm to reduce overhead efficiency. The experiment for this proposal needs to be performed using traffic and network simulators. Finally, the following is a brief overview of the paper’s most significant findings.

- The proposed novel architecture concept for 5G-enabled vehicle networks based on fog computing. The goal of this new layout is to boost the safety, confidentiality, and efficiency of existing vehicular networks.
- For 5G-enabled vehicle networks, this study offers a CPPA system based on fog computing; we term it the FC-CPPA scheme.
- We suggest using a fog server in conjunction with the FC-CPPA scheme to produce and preload a set of public anonymity identities and the related signature keys to each genuine vehicle.
- To meet the needs of privacy and security, we present a proof of the robustness of the proposed FC-CPPA method, noting the DDH problem’s hardness in the random oracle model.
- We present the performance of the proposed FC-CPPA scheme in terms of communication and computation costs, which are more efficient in message signing and single and batch signature verification as compared to related work.

## Figures and Tables

**Table 1:** Analysis of Current Authentication Systems’ Security.

	Malhi and Batra [19]	Jiang et al. [20]	Azees et al. [21]	Wu et al. [23]	FC-CPPA Scheme
Mutual Authentication	Yes	NO	NO	NO	Yes
Integrity	Yes	Yes	Yes	Yes	Yes
Anonymity Privacy-Preserving	Yes	Yes	Yes	Yes	Yes
Unlikability	Yes	Yes	Yes	Yes	Yes
Traceability	Yes	Yes	Yes	Yes	Yes
Revocability	Yes	Yes	Yes	Yes	Yes
Replaying Resistance	Yes	Yes	NO	Yes	Yes
NO RSU aided	NO	NO	NO	NO	Yes

**Table 2:** Software and Hardware Specifications.

Hardware	Features
Windows	Windows 11 professional
CPU	AMD Ryzen 7 5800H
RAM	8.00 GB
Architecture	64-bit CPU

**Table 3:** A Comparison of the Times Needed for Common Cryptographic Operations.

Cryptography Operation	Notation	Running Time (ms)
The amount of time required to form a bilinear pair in $G_{1}$	$P_{bp}$	5.811
The amount of time required to form a scalar multiplication operation in $G_{1}$	$M_{bp}$	1.5654
The amount of time required to form a point addition operation in $G_{1}$	$A_{bp}$	0.0106
The amount of time required to form a map-to-point hash function in $G_{1}$	$H_{mtp}$	4.1724
The amount of time required to form a scalar multiplication operation in *G*	$M_{ecc}$	0.6718
The amount of time required to form a point addition operation in *G*	$A_{ecc}$	0.0031

**Table 4:** Five Authentication Methods and Their Relative Computation Costs.

Scheme	MsgSign Phase (ms)	SigVerify Phase (ms)	BSigVerify Phase (ms)
Azees et al. [21]	$4M_{bp}\approx 2.6872$	$2P_{bp}+5M_{bp}+2A_{bp}\approx 19.4702$	$\left(n+1\right)P_{bp}+5nM_{bp}+2nA_{bp}\approx 13.6592n+5.811$
Al-Shareeda et al. [22]	$1M_{bp}\approx 1.5654$	$2P_{bp}+2M_{bp}+1A_{bp}\approx 6.2722$	$P_{bp}+2nM_{bp}+nA_{bp}\approx 3.1414n+5.811$
Asaar et al. [24]	$7M_{ecc}\approx 4.7026$	$12M_{ecc}+8A_{ecc}\approx 8.0864$	$\left(4n+10\right)M_{ecc}+\left(6n+2\right)A_{ecc}\approx 2.7058n+6.7242$
Li et al. [25]	$1M_{ecc}\approx 0.6718$	$4M_{ecc}+1A_{ecc}\approx 2.6903$	$\left(2n+2\right)M_{ecc}+nA_{ecc}\approx 1.3467n+1.3436$
Alshudukhi et al. [26]	$2M_{ecc}+1A_{ecc}\approx 1.3467$	$3M_{ecc}+1A_{ecc}\approx 2.0185$	$\left(n+2\right)M_{ecc}+\left(n-1\right)A_{ecc}\approx 0.6749n+1.3405$
FC-CPPA	$1A_{ecc}\approx 0.0031$	$3M_{ecc}+1A_{ecc}\approx 2.0185$	$\left(n+2\right)M_{ecc}+\left(n-1\right)A_{ecc}\approx 0.6749n+1.3405$

**Table 5:** The Comparison of Communication Cost.

Scheme	Message-Signature Tuple	Size (Bytes)	n Size (Bytes)
Azees et al. [21]	$\left(Cert_{k},sig,Y_{k}\right)$	128 × 6 + 20 × 3 + 20 = 848	848n
Al-Shareeda et al. [22]	$\left(pid_{i}^{1},pid_{i}^{2},m_{i},svt,ts,\delta_{m_{i}}\right)$	128 + 2 × 20 + 2 × 4 = 216	216n
Asaar et al. [24]	$\left(PID_{i},T_{i},m_{i},R_{i},W_{i},s_{i,1},s_{i,2}\right)$	40 × 3 + 20 × 3 + 4 = 184	184n
Li et al. [25]	$\left(M_{i},PID_{i,l},PK_{i,l},R_{i},T_{i},sig_{i}\right)$	40 × 3 + 20 + 4 = 144	144n
Alshudukhi et al. [26]	$\left(PsID_{i}^{1},PsID_{i}^{2},m_{i},TS_{i},\sigma_{m_{i}}\right)$	40 + 2 × 20 + 4 = 84	84n
Our Proposed	$\left(SMsg_{i},PAID_{i}^{1},PAID_{i}^{2},ts_{i},\sigma_{Veh_{i}}\right)$	40 + 2 × 20 + 4 = 84	84n

## Data Availability

Not applicable.
